# Fingerprints and Floor Plans Construction for Indoor Localisation Based on Crowdsourcing

**DOI:** 10.3390/s19040919

**Published:** 2019-02-22

**Authors:** Ricardo Santos, Marília Barandas, Ricardo Leonardo, Hugo Gamboa

**Affiliations:** 1 Associação Fraunhofer Portugal Research, Rua Alfredo Allen 455/461, 4200-135 Porto, Portugal; marilia.barandas@fraunhofer.pt (M.B.); ricardo.leonardo@fraunhofer.pt (R.L.); hugo.gamboa@fraunhofer.pt (H.G.); 2 Laboratório de Instrumentação, Engenharia Biomédica e Física da Radiação (LIBPhys-UNL), Departamento de Física, Faculdade de Ciências e Tecnologia da Universidade Nova de Lisboa, Monte da Caparica, 2829-516 Caparica, Portugal

**Keywords:** indoor localisation, fingerprinting, crowdsourcing, indoor mapping, floor plan construction, pedestrian dead reckoning, unsupervised machine learning, time series similarities

## Abstract

The demand for easily deployable indoor localisation solutions has been growing. Although several systems have been proposed, their limitations regarding the high implementation costs hinder most of them to be widely used. Fingerprinting-based IPS (Indoor Positioning Systems) depend on characteristics pervasively available in buildings. However, such systems require indoor floor plans, which might not be available, as well as environmental fingerprints, that need to be collected through human resources intensive processes. To overcome these limitations, this paper proposes an algorithm for the automatic construction of indoor maps and fingerprints, solely depending on non-annotated crowdsourced data from smartphones. Our system relies on multiple gait-model based filtering techniques for accurate movement quantification in combination with opportunistic sensing observations. After the reconstruction of users’ movement with PDR (Pedestrian Dead Reckoning) techniques, Wi-Fi measurements are clustered to partition the trajectories into segments. Similar segments, which belong to the same cluster, are identified using an adaptive approach based on a geomagnetic field distance. Finally, the floor plans are obtained through a data fusion process. Merging the acquired environmental data using the obtained floor plan, fingerprints are aligned to physical locations. Experimental results show that the proposed solution achieved comparable floor plans and fingerprints to those acquired manually, allowing the conclusion that is possible to automate the setup process of infrastructure-free IPS.

## 1. Introduction

The dissemination of smart mobile devices, especially in the last decade, has supported the development of location-based services, which acquired a major role in our daily life. While the GPS (Global Positioning System) is the standard solution for positioning and navigation outdoors, its accuracy is compromised indoors, where the presence of walls and ceilings attenuates the satellite signals.

Considering this problem, several IPS (Indoor Positioning Systems) have been proposed [1,2,3,4,5,6,7,8,9], where alternative sources of information are leveraged. Infrastructure-based solutions require the installation and maintenance of equipment, commonly beacons, to produce artificial signals to be processed. On the other hand, infrastructure-free solutions are based on two major approaches, range-based or fingerprinting-based. Range-based approaches use the distance estimation between reference points and a target object. However, due to signal reflections, non-line-of-sight propagation and object movement, these approaches usually suffer from either low positioning accuracy or high hardware and computational costs [10]. Fingerprinting-based solutions rely in opportunistic readings from signals that are pervasively available in the majority of environments, which lowers their implementation costs.

Nevertheless, this last type of system requires an extensive process to collect the updated buildings information, which may hinder its practical implementation, especially in large-scale applications. Fingerprints that store the buildings’ environmental characteristics, such as the local magnetic field or the Wi-Fi distribution patterns, must be mapped, so that they can be used to locate the device. Furthermore, indoor floor plans are also often essential in these systems, where the layout of the building is registered to ensure the necessary accuracy of the positioning. However, indoor floor plans are not always available, potentially requiring their creation.

The traditional process of constructing floor plans requires the presence of professionals to map the features of the buildings, specifying with precision the dimensions of every room, for example [11]. The mapping of environmental fingerprints is also performed by specialists, who walk through the whole building to collect the data to be used [1]. Both processes are labour-intensive and time-consuming, especially in large buildings, such as malls, hospitals or airports. Moreover, some buildings present dynamics that change their environmental characteristics, as fairs and some shopping areas, requiring recurrent data collections to reconstruct the floor plans and remap the fingerprints. Thus, the search for a easily deployable and scalable solution remains open.

To overcome the aforementioned limitations of current IPS, in recent times, crowdsourcing is being suggested as a way to create self-sustaining systems, which do not require almost any specific human intervention. Crowdsourcing is defined as a contribution model, where a group of anonymous and voluntary people is called to help in solving a problem, developing a task or reaching a goal [12]. The volunteers are motivated to give their contribution in exchange of incentive mechanisms, as for entertainment, money or some service [13]. Several companies and organisations use crowdsourcing in their applications, such as Waze (www.waze.com), an outdoor navigation mobile app, that provides real time traffic information with data collected by their users. Collaborative translation and crowdfunding can also be classified as crowdsourcing applications.

In the indoor location field, crowdsourcing can be used in an opportunistic way, where non-annotated data is collected from the users’ smartphones, such as they naturally move throughout the buildings [12]. This data is then processed to obtain the buildings’ floor plans and/or their environmental fingerprints. Then, by continuously receiving data from different users, the floor plans and fingerprints can be progressively improved [2].

Taking into consideration the limitations of current IPS and the potentialities of crowdsourcing, we present an innovative algorithm to automatically construct indoor floor plans and environmental fingerprints. It only relies on data collected in a non-annotated way with smart mobile devices, through crowdsourcing-similar techniques. Since we use a large amount of data, collected opportunistically by several users during their usual activities inside buildings, this solution is ready to be applied in crowdsourcing indoor localisation applications. As incentive mechanisms, the users can benefit from the positioning service, while they collect more data to improve the floor plans and fingerprints. Leveraging the inertial and environmental sensors widely available in common smartphones, we are able to reconstruct the users’ trajectories, which carry information about the layout of the buildings. Then, the evaluation of the unique interference patterns on the buildings’ magnetic field, as well as the special features of the Wi-Fi networks, deployed in most of today’s buildings, allows the identification of similarities between the collected data. Our algorithm is then able to merge all the similar data, obtaining an accurate approximation of the buildings’ floor plans and environmental fingerprints, similar to those collected with traditional methods.

The main contributions of this novel unsupervised approach will ease the setup phase of fingerprinting-based IPS. Therefore, we are able to offer a solution that:

- Uses unlabelled data, collected opportunistically with crowdsourcing-based techniques, from a large amount of users.
- Effortlessly constructs the often unavailable buildings’ floor plans.
- Collects the environmental data and maps the fingerprints, required in most fingerprinting-based solutions.
- Is able to be widely deployed, since it uses data pervasively available in most buildings.
- Is not constrained to buildings with specific characteristics, as with a minimum area or without any open spaces.

With the contributions of our work, we are able to extend the range of scenarios where location-based services can be applied indoors.

In the following sections, we discuss the previous approaches regarding this topic (Section 2) and describe the architecture of the developed algorithm (Section 3). Finally, the results obtained in the system evaluation are presented (Section 4) and the taken conclusions are drawn (Section 5).

## 2. Related Work

Since the last decade, the effort from the scientific community to overcome the implementation limitations of fingerprinting-based IPS has resulted in a large number of solutions. Aiming to diminish the required human effort, these solutions often use crowdsourced data to automatically construct indoor floor plans and/or map environmental fingerprints, relying on several sources of information. Most of existing solutions only address one of the problems, still demanding human effort to implement parts of the system. Other solutions intend to promote completely autonomous systems, but present restrictive limitations.

Regarding the solutions that automatically construct indoor floor plans with pervasively collected data, they often rely on the inertial processing to infer the users’ trajectories. However, the noise accumulation affects the movement reconstruction, creating progressively escalating errors. Therefore, some of these solutions process alternative data sources. Walkie-Markie [14] relies on the Wi-Fi infrastructures available in most buildings to identify landmarks, the locations at which the signal trend on an AP (Access Point) reverses, being used to merge the large volumes of data and to correct the drift errors. SmartSLAM [15] also uses the Wi-Fi signal patterns combined with SLAM (Simultaneous Localisation and Mapping) techniques [16], to gradually construct the indoor floor plans while tracking the users movement. However, it is only able to map the main areas of the buildings, such as the layout of corridors, annotated with the Wi-Fi measurements. On the other hand, Luo et al. [11] rely on the special characteristics of the geomagnetic field, together with the inertial tracking, to construct indoor floor plans through an hierarchical clustering approach, by identifying similar magnetic sequences. Contrarily, CrowdInside [17] only processes inertial data, to not only reconstruct the users movement, but also to identify landmarks, which in this case consist on special signatures detected in the signals.

When considering the solutions that intend to reduce the effort on the fingerprints acquisition, the floor plan of the buildings is often required, which still might need to be constructed. Zee [18] is a system that reduces the effort of mapping Wi-Fi fingerprints, by tracking the users trajectories indoors. When combining the reconstructed movement and the floor plan constrains, it is possible to obtain the most probable last position of each trajectory. Finally, a backward propagation algorithm attributes the remaining path to the positions of the map, registering the collected data. Jung et al. [19] have developed a solution based on the same principle, where reconstructed trajectories with unlabelled fingerprints are fitted into the floor plan, to identify the corresponding positions. This algorithm has the great advantage of being prepared for the automatic update of fingerprints, to respond to the occasional changes in the buildings. LiFS [4] is an IPS that constructs the crowdsourced fingerprints by processing the floor plans with MDS (Multidimensional Scaling), to improve the match between the trajectories and the processed map.

Although most systems only address one of the problems, some solutions approach both. For example, PiLoc [2] performs clustering to segment the inferred trajectories. Then, the similar ones are identified and the maps and fingerprints are built.

Even though the high number of solutions that have been proposed, it is still needed a solution that is robust enough to satisfy all the needs. Therefore, we propose an innovative solution that surpasses the limited features of the previous systems. Instead of being restrained in the number of sources of information, we rely not only on the traditional inertial data, but also on the buildings’ Wi-Fi signal distribution and the local magnetic field pattern of interferences. By merging these layers of information, we eliminate the need of the buildings’ floor plans [18], or the need of the partial annotation of the data, done through the definition of an initial number of physical landmarks [3]. Furthermore, our solution is not restrained to the buildings dimensions, while others are limited to buildings with large corridors [2] or without large open spaces [14,18], for example. We are also able to reconstruct the floor plans, while others only map the main areas of the buildings [15]. For these reasons, we are able to surpass the limitations of the aforementioned methods by offering an autonomous solution that can be applied in existing IPS.

## 3. Algorithm

Although the number of solutions that apply crowdsourcing concepts to the indoor location field is increasing, their limitations still hinder them to be widely spread. Some solutions only address one of the problems of fingerprinting-based IPS, where the floor plans are constructed or the fingerprints are mapped. Others present limited accuracy, mainly due to the noisy sensors that accumulate errors through the several stages of the algorithms, or are restricted to specific types of buildings [2,14,18].

In order to overcome those limitations, we present an algorithm that constructs indoor floor plans and environmental fingerprints, without any specific effort from the users. To eliminate the effects of the error accumulation, mainly caused by the inertial sensors in the reconstruction of the movement phase, we combine the traditional PDR (Pedestrian Dead Reckoning) techniques with, not only the information retrieved from the geomagnetic field, but also from the Wi-Fi networks pervasively available in almost every building. Furthermore, unlike most of the current solutions, our system works without any restrains concerning the buildings’ dimensions and characteristics, which enlarges the applicability of autonomous IPS, where small environments, such as homes, can now be considered.

### 3.1. Architecture Overview

**Figure 1:**
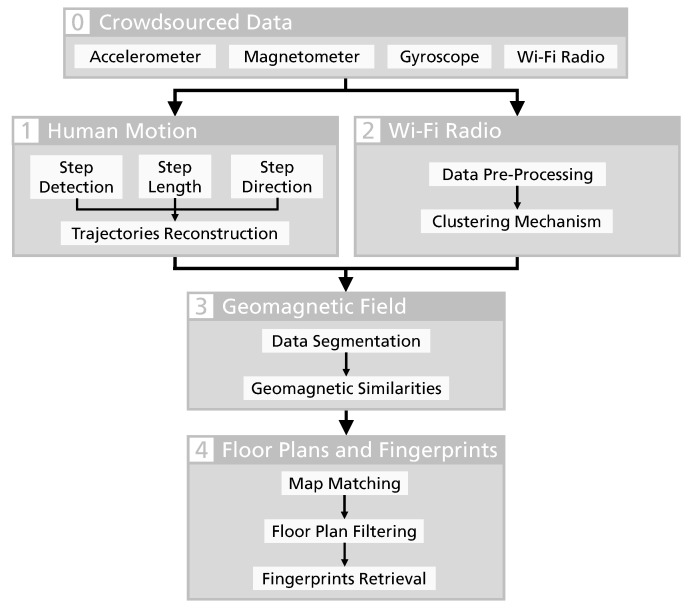
Architecture of the proposed algorithm for the automatic construction of indoor floor plans and environmental fingerprints.

Our algorithm can be divided into four main modules, as it can be understood in Figure 1. The algorithm processes the non-annotated data from the smartphones’ inertial sensors, such as the accelerometer, the gyroscope and the magnetometer. This is done to reconstruct the users’ trajectories through the identification of the human walking patterns, but also to detect similarities between the data collected during the inferred paths. To increase the system’s computational performance and to improve its results, we also collect information about the Wi-Fi networks available in the surroundings. However, considering that some buildings might not have these wireless networks, this information is not mandatory for the proper functioning of the system.

After receiving the crowdsourced data from the users’ smartphones, the following modules are processed sequentially:

1. Human movement reconstruction with PDR techniques, through steps detection and stride and heading estimation.
2. Wi-Fi measurements clustering with unsupervised machine learning techniques.
3. Geomagnetic data processing and identification of similarities.
4. Construction of indoor floor plans and mapping of environmental fingerprints, as a result of the fusion of users’ trajectories and filtering operations.

The following sections address the process and each of the four main modules that characterise the presented algorithm, as depicted in Figure 1.

### 3.2. Human Motion

In the first module of our algorithm, the data collected from the embedded inertial sensors is used to infer the human movement with PDR techniques. This process is an essential requirement to our solution, since it is the reconstruction of the trajectories that allows the construction of floor plans, through the identification of similarities between different users’ trajectories.

#### 3.2.1. Trajectories Reconstruction

The trajectories reconstruction is obtained through the identification of human steps and their corresponding length and direction. A sensor fusion algorithm is implemented to minimise the variations caused by the orientation of the smartphones in relation to the body, but also the imprecisions of the estimation of the magnetic North direction indoors, from the magnetometer [5]. The orientation of the device relative to the Earth reference frame is identified by merging data from the inertial sensors, using a second order complementary filter [1]. Combining the long-term reference to the gravity direction, from the accelerometer, and to the North, from the magnetometer, together with the short-term accuracy of the angular rotation, from the gyroscope, it is possible to orient the smartphones in relation to the Earth frame. With this, we are able to discriminate the accelerations caused by the movement, from the acceleration caused by the gravity [1].

Accordingly to the study conducted by Zijlstra et al. [20], it is possible to infer the characteristics of the movement with the evaluation of the trunk acceleration data while walking. Since the human movement can be described with a gait model, where each step performed by a user produces cyclic patterns on the acceleration, it is possible to identify the instants when the users’ touch the floor, by the distinct peaks created on the vertical acceleration. To do so, we apply a low pass filter to remove the noise and then a combination of adaptive threshold methods and a decision tree classifier.

Once a step is detected, its length, $l_{s}$, is estimated by the Weinberg’s method [21], which is based on an empirical relationship between the step length and the difference between the maximum and minimum peaks of the vertical acceleration:

(1) $$l_{s}=K\cdot \sqrt[{4}]{A_{max}-A_{min}}$$

where $A_{max}$ and $A_{min}$ are the maximum and minimum vertical accelerations of a step, respectively. *K* is a calibration constant, recursively adjusted by the least-squares method.

Too characterise the human movement, it is necessary to determine the relative orientation of consecutive steps. Since the computation of the absolute orientation of the user in relation to the magnetic North cannot be obtained with high accuracy, due to magnetic interferences [5], we process the gyroscope data to obtain the relative changes in the movement heading. By integrating the vertical component of the gyroscope in the Earth frame, ${\dot{\theta }}_{t}^{z}$, we obtain an angle that represents the heading of the smartphone, $\theta_{t}$, in relation to the heading of a previous instant, $\theta_{t-1}$, through the result of:

(2) $$\theta_{t}=\theta_{t-1}+{\dot{\theta }}_{t}^{z}\Delta t-\varepsilon_{d}$$

Since typical consumer-grade gyroscopes present a significant bias error, this integration will cause the inferred trajectories to drift. To minimise this effect, we apply a threshold-based approach to determine whether a user is turning or walking straight. After the determination of the observed drift during a straight line walk, $\varepsilon_{d}$, we refine the heading calculations to precisely obtain the relative variation of the user direction between steps.

After determining all the necessary parameters to characterise the movement, we reconstruct the trajectories by identifying the consecutive positions after each step in a two-dimensional plane. By placing the initial position of each trajectory at the origin of the plane, we compute the subsequent position through the results of:

(3) $$\left(x_{s},\;y_{s}\right)=\left(x_{s-1},\;y_{s-1}\right)+\left(l_{s},\;0\right)\cdot \left[\begin{array}{cc} \cos\theta_{t} & -\sin\theta_{t}\\ \sin\theta_{t} & \cos\theta_{t} \end{array}\right]$$

where the achieved position $\left(x_{s},\;y_{s}\right)$, after a step *s* was taken, depends on the position of the previous step, $\left(x_{s-1},\;y_{s-1}\right)$, as well as the length of the new step, $l_{s}$, and its corresponding direction, $\theta_{t}$.

#### 3.2.2. Domain Conversion

Human motion in natural environments is complex due to the large variability of walking patterns. For example, two people walking throughout a building, such as an elderly and a young adult, display different walking speeds. To cope with this misalignment in time, a distance domain conversion is applied to the environmental signals (geomagnetic field and Wi-Fi). In other words, instead of having the signals indexed to a certain instant, they will vary accordingly to the distance travelled in each trajectory. Since we have information about the timestamps of every step, as well as their corresponding length, we are able to retrieve the data that was acquired at a given travelled distance, through a linear interpolation.

**Figure 2:**
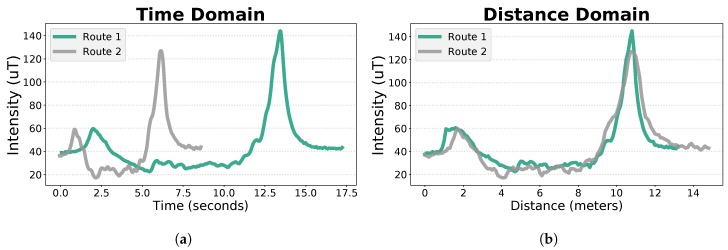
Results of the magnetic signal domain conversion. In (**a**), the magnitude of the magnetic field for two routes collected through the same path, by two different people, are represented in the time domain. In (**b**), the signals of the same routes are represented in the distance domain, after the domain conversion process, as it can be denoted by the interference pattern alignment.

Independently from the collection device, the magnetic field signals have a high sampling rate. Thus, we are able to choose any fixed step value, from which the corresponding data points will be retrieved. Although we could choose any step value, it is important to guarantee that we have enough data points to accurately represent the signals, but it is also essential to ensure that we do not exceed the necessary points, which would increase the computational complexity. So, we defined the step value as 10 cm. After this process, the geomagnetic field of two routes acquired in the same location, by two different people, can be directly compared. As it can be seen in Figure 2a, the magnetic signals of two routes acquired in the same location are represented in the time domain, while in Figure 2b the same signals were converted, being displayed in the distance domain.

Regarding the Wi-Fi measurements, where the APs’ replies are received in packages, having sampling rates lesser than 1 Hz, an alternative process of conversion is considered. Since we cannot obtain a data point for a fixed distance step, the timestamp of each Wi-Fi reply package is instead converted to a distance, through a linear interpolation between the preceding and following steps. With this process, we obtain each reply package indexed by a distance, instead of an instant.

### 3.3. Wi-Fi

The second module of our solution includes the processing of the collected Wi-Fi measurements, followed by its evaluation with a clustering technique. Even though this module is not indispensable to construct the indoor maps and fingerprints, it has a significant role on assuring the quality of the results, being also an important factor in lowering the computational costs of the algorithm.

Wi-Fi networks are being used in several fingerprinting-based solutions [1,5,6,7,8,9], supported not only by the wide spread of wireless networks indoors, but also for the signal’s properties. Due to the presence of interferences in the Wi-Fi signal caused by the buildings’ infrastructures, our algorithm deals with typical errors by applying different mechanisms before the clustering process.

The Wi-Fi measurements comprise the information about BSSIDs (Basic Service Set Identifier) detectable from the device at each scan, as well as the information of all WLANs (Wireless Local Area Networks) that they have defined at each radio band (2.4 and 5 GHz), together with the perceived RSSI (Received Signal Strength Indicator). After the pre-processing, the collected data packages from the APs replies, which are the objects to be clustered, are evaluated and different labels are attributed, depending on the final number of clusters. Since the objects of the same cluster have very similar information, it is possible to infer that they were collected in the same area, due to the characteristics of the Wi-Fi networks, such as the uniqueness of each BSSID and the signal’s strength decay pattern.

#### 3.3.1. Data Pre-Processing

The Wi-Fi measurements pre-processing is done not only to extract the required features for the clustering algorithm, but also to diminish the possibilities of error in the final results, related to the identified issues of this signal [9], that often compromise the accuracy of several solutions. In this sense, the identification of reliable APs for localisation purposes, followed by their signal strength processing for features computation is performed.

The identification of the most reliable APs for the positioning phase consists on a search through the entire data to identify all APs ever detected in the building. In order to eliminate unreliable APs, as smartphone hotspots, that would add dissimilarity to Wi-Fi signals collected in the same place, only APs that emit more than one WLAN are considered.

Regarding the Wi-Fi signal strength processing, the RSSI values from the same AP, collected in the same data package, are merged into a single value using the mean of all WLANs’ RSSIs. Considering that RSSIs below −100 dBm are usually undetectable by most devices, we consider this value as the lower limit of the accepted strength values’ range. With the purpose of standardising the data, APs that are missing in a set of received Wi-Fi measurements will have their signal strength value equals to −100 dBm. Since not all devices support the 5 GHz radio band, we only consider, for the clustering process, the Wi-Fi replies of the WLANs that emit within the 2.4 GHz radio band. With this process, the data collected by different smartphones will have the same characteristics, not influencing the clustering results.

With the packages of Wi-Fi replies processed, the clustering mechanism could be directly applied, where the features would be the RSSI values of the detected APs. However, we verified that signals collected by different smartphones in the same locations vary, which introduce differences in the features depending on the collection device. Therefore, we defined the features of each set of Wi-Fi replies as the RSSI differences between every pair of APs, since the relative RSSIs of the APs between different devices are more consistent [22].

#### 3.3.2. Clustering Mechanism

In this work, the clustering mechanism will evaluate and label each object, i.e., each collected Wi-Fi reply package. The variety of clustering algorithms that have been proposed in the last decades imposes a careful study about the characteristics required for each context. Depending on the objective of the clustering process, some algorithms might be useful, while others may fail, even though they have proven results. Here, we aim to cluster the Wi-Fi measurements, collected in large quantities with crowdsourcing techniques.

With a high number of objects to be labelled, computationally heavy algorithms will not offer the scalability conditions that we propose. Hierarchical clustering is one of most common approaches, where the partition is achieved progressively through the agglomeration and division of clusters, considering their similarity. Until a stopping criteria is met, which can be hard to define in unknown datasets, the process continues and the similarity between all objects is recomputed to merge/divide the most similar/different ones. This iterative process increases the computational complexity of the clustering process, hindering this algorithm to be applied in large datasets.

Furthermore, the number of dimensions of the feature space, χ, grows quadratically with the number of detected APs in the building, being expressed by:

(4) $$\chi =\frac{n^{2}-n}{2}$$

where *n* is the number of APs in the building. Thus, clustering algorithms that require the definition of parameters depending on the evaluation of the feature space can hardly be applied. For example, DBSCAN [23], a density-based algorithm that has been showing promising results, for its capability of detecting outliers, requires the previous determination of Eps, the minimum distance between two objects to be clustered together. With a high number of dimensions, determining the optimum value for this parameter is non-trivial task. Secondly, in DBSCAN, the minimum number of objects in a cluster, MinPts, also has to be defined. Based on the assumption that the constitution of the database for different buildings varies, where we cannot previously infer the expected density of a cluster, DBSCAN is not the ideal approach to our goal. Although HDBSCAN [24] resorts to a hierarchical approach to determine MinPts, the definition of Eps remains a problem.

Taking into account the limitations that both the hierarchical and the density-based approaches present, as well as the features that the partitional approach offers, we selected this last one to apply in our solution. K-Means is the most well-known partitional clustering algorithm [25]. It works well in large datasets and has the advantage of being able to adjust the cluster distributions until an optimal point is reached. As K-Means assigns each object to the closest centroid, it tends to produce globular clusters, neglecting the presence of outliers.

After the evaluation of the pros and cons of K-Means, we considered it as the best approach to our purposes. However, partitional algorithms require the definition of the number of clusters as input. Considering that we aim to develop an autonomous solution, with the minimum human interaction possible, a secondary process was implemented to automatically estimate the ideal number of clusters. Firstly, an evaluation graph is computed with the result, for a different number of total clusters, of the sum of the squared distances between each object and its cluster centroid, $sum_{within}$, given by Equation (5):

(5) $$sum_{within}=\sum_{k=1}^{K}\sum_{i\in S_{k}}\sum_{j=1}^{p}{\left(x_{ij}-{\bar{x}}_{kj}\right)}^{2}$$

where *K* is the number of clusters of each iteration, *i* represents each object being clustered, and *j* each feature of the feature space *p*. Usually, methods that aim to estimate the ideal number of clusters, try to recognise the elbow of the evaluation graph, which represents the point where adding another cluster to the process does not significantly improve the results. However, in order to avoid the introduction of human effort, we further implemented the concept deployed by Zhang et al. [26], which identifies the ideal number of clusters at the point in which the function given by the curvature of the evaluation graph, given by Equation (6), is maximum.

(6) $$\kappa =\frac{\left|y^{\prime \prime }\right|}{{\left(1+y^{\prime 2}\right)}^{3/2}}$$

where *y* represents the previously computed evaluation graph.

**Figure 3:**
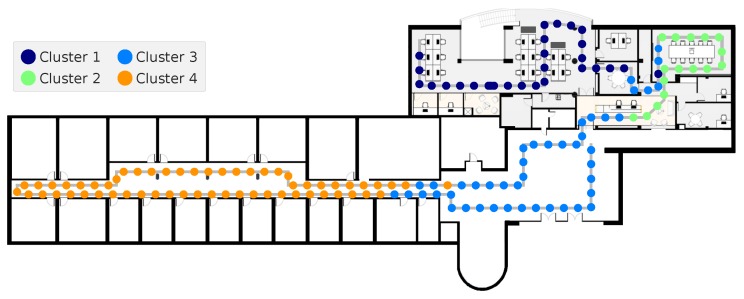
Example of the clustering distribution of the APs (Access Points) replies packages, for a route collected throughout a building.

To understand the output of this module, Figure 3 has represented a route that was described throughout the building below. A smartphone collected the Wi-Fi data, being each package of APs replies depicted by a circle. The position of each package was obtained with an annotation process, where the timestamp of each turn was registered. Then, with a linear interpolation, the approximate positions of the timestamp for all packages were computed. As it can be seen, the clustering process assigned each package with a different label, depending on the final clustering distribution. With these results, although there are some non-coherent Wi-Fi packages, it is possible to divide the building into four main areas.

### 3.4. Geomagnetic Field

The third module of this algorithm compares the geomagnetic data to identify similarities between users’ trajectories, which are expected to happen between segments of data that were collected in the same location.

Although the magnetic field remains naturally stable around the same area, its pattern is highly affected indoors [11]. The presence of metallic construction materials and electrical equipment causes disturbances in the geomagnetic pattern, which commonly produce unique patterns that can be used to identify a specific location.

#### 3.4.1. Data Segmentation

The process of identifying similarities between routes, by comparing the geomagnetic data, increases quadratically the complexity of our algorithm, since all collected magnetic field signals must be compared to each other, considering every possible alignment. To avoid this problem, the magnetic data is subject to the following segmentation process, to reduce the number of comparisons:

1. Users’ trajectories segmentation based on clustering process.
2. Straight lines identification using the human movement reconstruction.
3. Filtering of straight line segments that belong to the same cluster based on their length.

At first, with the results of the clustering module, where Wi-Fi signals are labelled into different clusters, it is possible to segment the users’ trajectories into smaller sections that belong to the same cluster, i.e., a user’s trajectory is divided into sections based on clustering process. Given that each cluster represents a specific area of the building, we can assume that the segmented sections of the same cluster were collected in nearby locations. Therefore, the identification of geomagnetic similarities is only done between trajectories sections of the same cluster.

The human movement reconstruction, obtained from the inertial processing, also gives useful information for easing the complexity of the algorithm. The similar segments that we aim to find in this module will be composed by pairs of segments that were acquired in the exact same locations of a building. Therefore, we are only interested in comparing segments that have similar inertial behaviour, as two straight segments or two turns. Having this in mind, and the fact that a reconstructed turn does not give much certainty, since it could be originated by a sudden tilt on the smartphone, we will retrieve from each trajectory the portion that corresponds to straight line segments, i.e., when the heading estimation between consecutive steps remains constant. Thereby, the obtained segments are composed by straight lines that belong to a unique section, previously identified by the clustering process.

Finally, we apply a cleaning process to remove the straight line segments that are smaller than a defined size, based on the fact that longer similar segments provide more confidence in assuming that they are actually in the same location. For example, it is more likely that two similar segments with 10 m have a unique magnetic field interference pattern, than two similar segments of only one meter, since their shorter patterns might be included in the interference patterns originated by multiple sources. Thus, a minimum of five meters is defined for each segment.

After this process, the complexity of the search for geomagnetic similarities is reduced, as well as the possibility of comparison errors.

#### 3.4.2. Similarities Identification

As it was already mentioned, the identification of similarities is done by comparing the geomagnetic field of the previously segmented sections. Since sections labelled with different clusters were acquired in different areas of the building, we will only be interested in performing comparisons between pairs of straight line segments that were clustered together.

**Figure 4:**
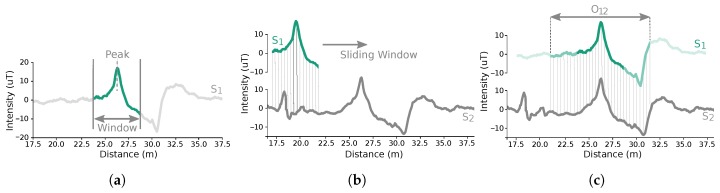
Overlap identification process, between segments $S_{1}$ and $S_{2}$. In (**a**), a window around the maximum peak of segment $S_{1}$ is retrieved and compared in (**b**) with the segment $S_{2}$, through a sliding mechanism. Then, in (**c**), the overlap $O_{12}$ is identified by the alignment that gave the higher similarity. A further process is applied, where the window is iteratively extended to verify the length of the overlap, until the limits of the segments are reached or until a dissimilarity is found.

The identification is done through an adaptive approach that uses a distance measure to compare pairs of segments, as illustrated in Figure 4. With the data available in the distance domain, we are able to apply lock-step measures, which compare two time series directly, where the *i*th point of the first is compared to the *i*th point of the second [27]. Since the obtained segments have different lengths, depending on the performed trajectory, we apply a sliding window mechanism. Given that the useful characteristics of the magnetic field are the interferences on its stable pattern, the window to be slid is centred around the maximum identified peak on the magnetic field magnitude of each segment (Figure 4a). Then, the distance between the window and each alignment of the segment candidate (Figure 4b) is computed by combining each magnetic field component, through the WPA (Windowed P-norm Alignment) measure [28], defined in Equation (7):

(7) $$dist_{i}=\frac{\sum_{j=1}^{N}{\left(\prod_{a=1}^{3}{\left|sig_{a{\left[i:i+N\right]}_{j}}-w_{aj}\right|}^{p}\right)}^{1/p}}{N}$$

where $dist_{i}$ stores the distance for each alignment i∈[0,l−N+1], with *l* the length of the segment candidate, sig, and *N* the length of the window, *w*. $dist_{i}$ is computed through the combination of the differences between the corresponding portions of the segment candidate, $sig_{\left[i:i+N\right]}$, and the window, *w*, for each one of the three magnetic field components, *a*. The norm *p* was defined as 1. When the global minimum distance is below a defined similarity threshold, an overlap is identified and the window is extended (Figure 4c) through an iterative process, to determine the length of the overlap. It consists on the recomputation of the WPA measure, where at each iteration the window is extended by 10% of the initial length to both sides, until the limits of the shorter segment or until the global minimum distance stays above the similarity threshold. The similarities identification process is repeated with the inverted segments, based on the assumption that two routes can be acquired in the same place, but with two subjects walking in opposite directions. Thus, an overlap is composed by two similar segments, where the global minimum distance value, $dist_{min}$, and the maximum achieved window length, $N_{max}$, are annotated, so they can be used to construct the floor plans.

The similarity threshold was empirically determined as 0.005, being low enough to avoid the identification of mistaken overlaps. Still, considering the large amount of data that is inherent to crowdsourcing techniques, it is preferable to only use the overlaps in which that we have certainty that they are correct, thus hindering the floor plan reconstruction error accumulation, already caused by the noisy inertial sensors.

### 3.5. Floor Plan and Fingerprints Construction

The final module of our algorithm relies on the previously identified overlaps to construct the indoor floor plans, as well as mapping the environmental fingerprints.

#### 3.5.1. Map Matching

Using the reconstructed users’ trajectories, collected during their natural moving across the buildings, the preliminary floor plans are constructed, through a process named map matching. This process progressively constructs the floor plans by adding new routes to the map, transforming their coordinate system into the coordinate system of the ones that were already mapped.

A map is defined as a bi-dimensional matrix, where each cell represents a physical location, and stores the number of routes that were mapped into that position. The resolution of the map is defined to be of one square meter, since that precision is enough in the majority of the indoor location applications and is aligned with the accuracies of the available solutions [29]. To match this requirement, each trajectory is transformed to the new resolution.

As mentioned before, a user’s trajectory can be composed by straight line segments that belong to the same cluster. Thus, each segment of a user’s trajectory can match with several segments from different users’ trajectories, since several routes can pass by the same corridor. Moreover, each user’s trajectory can also match with other users’ trajectories in several different segments. Considering this, and to avoid the rise of the computational costs of the algorithm, each route will only be mapped once, through the identification of the overlap that achieved the highest similarity index, given by:

(8) $$similarity_{index}=\frac{N_{max}}{dist_{min}}$$

weighting the global minimum distance, $dist_{min}$, proportionally to the maximum window length achieved, $N_{max}$. To better understand the map matching process, let us consider that an overlap $O_{12}$, composed by the segments $S_{1}$ and $S_{2}$ from routes $R_{A}$ and $R_{B}$, respectively, has the highest $similarity_{index}$. Firstly, route $R_{A}$ is placed in the centre of the map (bi-dimensional matrix) and next, route $R_{B}$ is transformed into the coordinate system of route $R_{A}$ through translation and rotation operations, in a way that $S_{1}$ and $S_{2}$ are overlaid in the map. Then, a search over the overlaps that contain segments $S_{1}$ and $S_{2}$ is done, and the same transformation process is applied to the new routes. When no more overlaps have neither of the segments already mapped, the search restarts to find the next overlap with the highest $similarity_{index}$, that has one of the routes already in the map, repeating the previous process. This mapping procedure occurs iteratively until there is no possibility to map more routes. At this point, a preliminary floor plan is available. However, if there is still overlaps to be processed, having neither of the routes already in the map, a new map is created and the process is restarted. When all overlaps are processed, among all created floor plans, the one that contains more data is selected as the final floor plan of the building, since it is considered to be the most complete. Nevertheless, with the large amount of data obtained with crowdsourcing, this problem tends to disappear.

#### 3.5.2. Floor Plan Filtering

The reconstructed floor plans have represented the shape of the buildings that were visited by their users. However, the errors originated by the inertial sensors and the great variability of the human movement affect the reconstruction, producing some blurred areas around the main zones. As we always want to produce a well-defined floor plan that can be used for the fingerprints mapping, we implemented a filtering mechanism.

Through the obtained floor plan, the median of the passages over all map positions is calculated. Using this information, all positions that store a number of passages equal or below the calculated median are eliminated, based on the consideration that they do not carry enough data. Since this process often originates sparse sets of positions, we apply a contour identification mechanism to remove them. With the final floor plan properly identified, a binarisation process is employed, generating a final map with the information of the existing areas of the mapped building.

#### 3.5.3. Fingerprints Retrieval

The last step of this module consists on the retrieval of the environmental fingerprints, to be used in common fingerprinting-based indoor location solutions. Depending on the system, different information sources can be used. Here, we focused on the two mainly used sources: the geomagnetic field and the Wi-Fi measurements. Nevertheless, the algorithm is capable of mapping fingerprints for different data, such as the Bluetooth or the sound pattern, introducing only the specific data processing that characterises each type, used in other approaches [30].

The fingerprints store the environmental readings on the constructed floor plan, in the same resolution of one square meter. For the geomagnetic field, fingerprints for all axes are obtained, while for the Wi-Fi, we produce as many fingerprints as the number of AP at each available radio band. Having the data in the distance domain, it is possible to interpolate the displacement of all data points to obtain the corresponding coordinates in the obtained floor plan. At each position, we perform the mean of values that were collected there, depending on the positioning of the routes in the constructed floor plan. This way, we generate robust fingerprints that have represented data from a wide variety of devices. This method surpasses one problem of the traditional collection methods, where a single device is used to map the fingerprints.

## 4. System Evaluation

**Figure 5:**
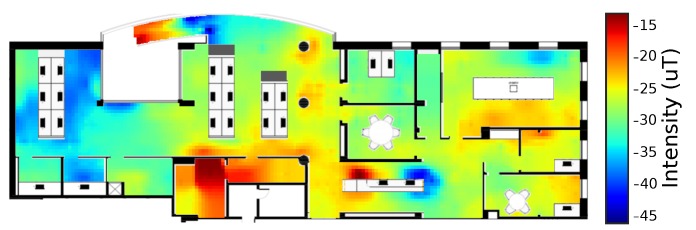
Original floor plan of the tested building, with the vertical component of the magnetic field fingerprint overlaid.

To validate the performance of our algorithm, we conducted an experiment in an office building. The floor plan of the tested building, with about 35 m by 12 m, as well as the corresponding magnetic fingerprint of the vertical component, collected by the traditional methods, are overlaid in Figure 5. The data used for the construction of the traditional magnetic fingerprint, present in Figure 5, was collected using a Samsung S6, through a technique described by Guimarães et al. [1].

For our system evaluation, data was acquired by six different people, who walked throughout the building, describing 22 different predefined routes. All testers held the smartphones in their hand and collected the data by walking at their natural speed. The acquisition is performed by a logger app, which opportunistically records the sensed data. The annotation of the walked paths was done, to allow the verification of the positioning accuracy with the constructed fingerprints. The users collected data with two different Android smartphones, a LG Nexus 5 and a Huawei Nexus 6P, in different days and in different times of the day, to ensure the variability of data that we want to consider in the final deployment of the system. For over 130 acquisitions, the users collected a total of 95 min of data.

### 4.1. Qualitative Evaluation

**Figure 6:**
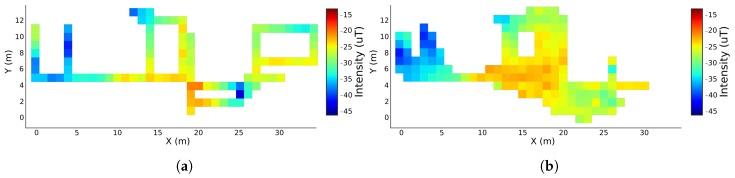
Magnetic field fingerprints for the vertical component. In (**a**), a squeletonised fingerprint collected with the traditional methods is represented. On the other hand, (**b**) has the fingerprint for the same component, but constructed with the proposed algorithm.

In order to ease the process of fingerprints comparison, a skeletonisation of the original magnetic fingerprints was done. For this skeletonisation, only the areas included in our predefined routes were considered. In Figure 6a, the result of this process, with a resolution of one square meter, is presented. Figure 6b shows the magnetic field fingerprint for the vertical component, constructed with the proposed unsupervised technique. Comparing both fingerprints, we can easily identify that the majority of the areas present similar magnetic field pattern. Although in the bottom values of the Figure 6b (Y lower than 4 m), the intensity values are attenuated when compared to the same area of Figure 6a, the variation pattern can still be identified.

**Figure 7:**
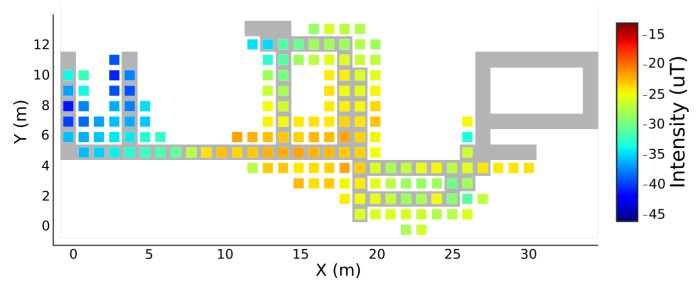
Original skeletonised floor plan, overlaid with the constructed fingerprint of the magnetic field vertical component.

For the floor plans comparison, Figure 7 shows the original skeletonised floor plan of Figure 6a, overlaid with the constructed fingerprint of Figure 6b. Through a comparison between both floor plans, an analysis of the results can be performed. Firstly, we can see that the building is well defined in the constructed floor plan, where most areas are accurately represented in terms of shape and dimensions. For example, the mapped corridors are well defined and the only case where two corridors were merged was in the inferior right corner of the floor plan, which can be explained by the fact that they are separated by less than one meter, gap that is actually lower than the resolution of the map. The right corner of the original floor plan was not mapped in the final floor plan, given that our predefined routes were mainly designed in the office open space. In our collection, only 15% of the acquisitions pass by the unmapped room. Considering that, in free living conditions, there are normal tilting movements that affect the heading estimation, introducing errors on the PDR reconstruction, it is expected that our algorithm does not consider some routes for the final map. Before the floor plan filtering, there were few routes matched in the unmapped room. However, the filtering process, described in Section 3.5.2, eliminated them. We assume that, in a larger dataset, this problem will not be verified. Furthermore, as it can be seen in Figure 6b, the corridors of our floor plan are not shown only by traces, but with the real dimensions where the users walk. The edges of the floor plan also show that the map is constructed with the real pattern of the human movement, since real changes of direction are not done by strict turns of 90º, for example, but with smooth and progressive changes. These outcomes place our algorithm in great advantage in relation to most existing solutions, which only deal with the main traces of the buildings, being limited in environments with special characteristics, as open spaces.

### 4.2. Quantitative Evaluation

#### 4.2.1. Constructed Floor Plan

Considering the positive results from the previous evaluation of the constructed floor plan, we performed some metrics, to quantitatively confirm the results. This evaluation was done by comparing the constructed floor plan, obtained from the binarisation of Figure 6b, with the original floor plan of the building, in the mapped areas, converted to a resolution of one square meter. To obtain the difference between both maps, we evaluated the floor plan construction as a binary classification problem, where a cell belonging to the map is considered as a positive, while the contrary is considered as a negative. Thus, we were able to perform some statistical metrics. In Table 1, the results of the computed metrics are available. By comparing the correctly constructed positions of the original map, given by the number of aligned one square meter cells, with the total number of positions in the constructed map, we were able to compute its precision. Then, the recall or sensitivity was obtained by comparing the same number of correctly identified positions of the original map with its total number of locations. Finally, the F1 score was computed to provide the certainty of the used tests, given by the harmonic average of the precision and recall.

The outcomes of these metrics confirm the results denoted in the qualitative evaluation. Furthermore, the false positives of the constructed map are present in the neighbouring areas of the existing corridors, which should not have a major impact in the positioning. With most of the areas of the original map correctly reconstructed, we can affirm that our solution is able to automatically construct indoor floor plans for fingerprinting-based IPS, as it will be confirmed in the further analyses.

#### 4.2.2. Environmental Fingerprints

**Figure 8:**
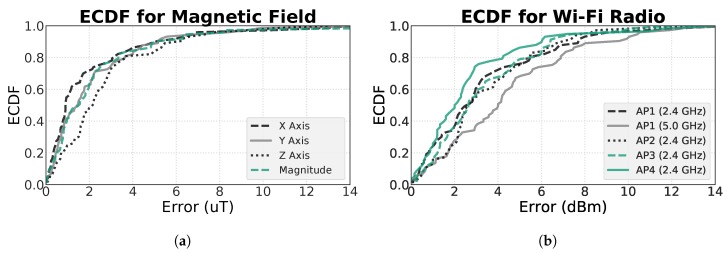
ECDFs (Empirical Cumulative Distribution Functions) for the errors in the constructed fingerprints. In (**a**), the ECDF is computed for each axis of the magnetometer, as well as the magnitude. In (**b**), a ECDF was computed for each AP, at each radio band.

In order to quantify the quality of the constructed fingerprints, we performed an evaluation over the environmental data values between the constructed and the original fingerprints. To do so, we used the skeletonised positions, obtained from the original floor plan, to compute the differences between the values of the matching positions in both fingerprints, which are available in Figure 7. In Figure 8, the ECDFs (Empirical Cumulative Distribution Functions) of the computed differences for all axes and magnitude of the magnetic field (Figure 8a), and for all reliable APs at each frequency band of the Wi-Fi (Figure 8b), are presented.

As it can be seen, in the magnetic field, the error does not surpass the 4 μT in the percentile 80 of every function, and at 50 percent of the points, the error is below 2 μT. On the other hand, the ECDFs of the Wi-Fi measurements also show great results, where the percentile 80 of the error of all APs is below 7 dBm. Furthermore, the mean error for the magnetic field points is 2.38 ± 2.86 μT, and for the Wi-Fi measurements is 3.6 ± 2.9 dBm. The results of this comparison show promising results, where the differences between both types of fingerprints are minimal, which is expected to not greatly affect the localisation results.

**Figure 9:**
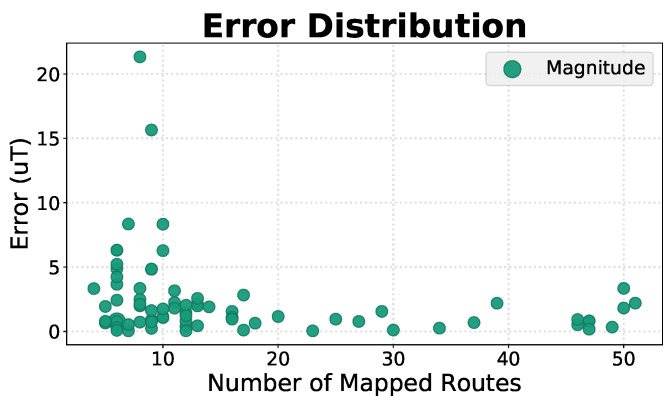
Distribution of the error of the magnetic field magnitude fingerprint, depending on the number of mapped routes in each position.

A further study was implemented, to evaluate the variation of the error depending on the number of routes that were mapped into each position. Figure 9 depicts the error distribution for all skeletonised positions, of the magnetic field’s magnitude, depending on the number of mapped routes. As it can be seen, the maximum error diminishes with the increase in the number of routes. This means that, with a larger dataset, compatible with the final application of the solution in a real scenario, the obtained results are expected to improve.

At last, the obtained fingerprints were tested in a fingerprinting-based IPS [1], which processes the Wi-Fi measurements and the magnetic field, to improve the positioning results. Since not all areas of the building were mapped in the constructed floor plan, only the data that was collected through the mapped areas was submitted to the test. Table 2 shows the mean in the positioning error of the tested routes, as well as the maximum error. The outcomes of this test are satisfactory, since the obtained positioning results for both types of fingerprints are very similar, which sustain the previous evaluation results. Although the errors for the constructed fingerprints are higher than the errors for the original ones, these values differ less than a meter. This difference does not affect the localisation purposes in most applications, as in navigation. Still, we expect that with more data, these values will approximate, or even surpass the results obtained with the traditional fingerprints.

## 5. Conclusions

This paper presented an innovative approach towards the automatic implementation of fingerprinting-based IPS, by taking advantage of the natural movement of people inside buildings. Relying on crowdsourcing techniques for the data acquisition, it is possible to eliminate the traditional extensive process of data collection, which consists on a laborious site survey to construct the floor plans and fingerprints. Avoiding these restrictive limitations, we were able to develop a solution that can be applied in any building, thus diminishing the gap between the potentialities of indoor and outdoor localisation.

Our algorithm employs an unsupervised approach to process different natures of data. Through PDR techniques, we are able to reconstruct the movement pattern of the users, the basis for the floor plans reconstruction. By processing the Wi-Fi measurements from the existing networks, we divide the buildings into smaller areas, easing the process of identifying overlaps between different users’ trajectories, done by applying similarity measures to the collected geomagnetic field. With the identified overlaps, the floor plans are then obtained by aligning the crowdsourced trajectories. Finally, we obtain the necessary environmental fingerprints when the collected data is interpolated with the new map positions. Using sensors that are available in most smartphones, our solution dismisses the installation of additional equipment.

The results that we achieved show that it is possible to obtain comparable floor plans, with fingerprints similar to those collected traditionally. Considering the localisation purposes, this error does not represent a problem, since the resolution is more than enough to accurately lead a person to the correct location.

## Figures and Tables

**Table 1:** Statistical metrics results, obtained from the comparison of the constructed and the original floor plan, for the test building.

Precision	Recall	F1 Score
67.1%	77.4%	71.8%

**Table 2:** Positioning results, obtained with both types of fingerprints. Each cell represents the mean and the standard deviation for the mean and maximum errors of all tested routes.

Positioning Results	Constructed Fingerprints	Original Fingerprints
Mean Error (m)	4.4 ± 2.0	3.7 ± 1.8
Maximum Error (m)	6.5 ± 2.6	5.7 ± 2.3
